# Accuracy of the phenotypic 1G test to detect *Mycobacterium tuberculosis* and drug resistance from sputa in the US-Mexico border

**DOI:** 10.1093/infdis/jiaf638

**Published:** 2026-04-29

**Authors:** Mia Aguirre, Doris Ayala, Juan Ignacio Garcia, Yoscelina E. Martinez-Lopez, Amberlee D. Hicks, Nadine Chacon, Ashley Gay-Cobb, Alyssa Schami, Selena Zavala-Perez, Ilse A. Dominguez-Trejo, America M. Cruz-Gonzalez, Raul Loera-Salazar, Javier E. Rodríguez-Herrera, Esperanza M. Garcia-Oropesa, Miryoung Lee, Adrian Rendón, Shu-Hua Wang, Marcel Yotebieng, Carlton A. Evans, Jordi B. Torrelles, Blanca I. Restrepo

**Affiliations:** 1 Department of Epidemiology, School of Public Health, University of Texas Health Science Center at Houston, Brownsville campus, Brownsville, TX, 78520, USA; 2 Population Health and Host Pathogens Interactions Programs Texas Biomedical Research Institute, San Antonio, TX 78229, USA; 3 International Center for the Advancement of Research & Education (I·CARE), Texas Biomedical Research Institute, San Antonio, TX 78229, USA; 4 Departamento Estatal de Micobacteriosis, Secretaría de Salud de Tamaulipas, Reynosa 88630, Matamoros 87370 and Ciudad Victoria 87000, Tamaulipas, México; 5 Unidad Académica Multidisciplinaria Reynosa-Aztlán, Universidad Autónoma de Tamaulipas 88740, Reynosa, México; 6 Centro de Investigación, Prevención y Tratamiento de Infecciones Respiratorias (CIPTIR) and Hospital Universitario “Dr. Jose Eleuterio Gonzalez”, 64460, Nuevo Leon, México; 7 Division of Infectious Disease, Department of Internal Medicine, College of Medicine, The Ohio State University, Columbus, OH 43210, United States; 8 Division of General Internal Medicine, Department of Medicine Albert Einstein College of Medicine, The Bronx, NY 10461, United States; 9 Innovation for Health And Development (IFHAD), Section of Adult Infectious Disease, Department of Infectious Disease, Imperial College London, South Kensington Campus, London SW7 2AZ, United Kingdom.; 10 Inovacion Por la Salud Y el Desarrollo, Asociacion Benefica PRISMA, 17073, Lima, Peru.; 11 Innovation for Health and Development (IFHAD), Laboratory of Research and Development, Faculty of Sciences and Engineering, Universidad Peruana Cayetano Heredia, 17073, Lima, Peru.; 12 South Texas Diabetes and Obesity Institute and Department of Human Genetics, School of Medicine, University of Texas Rio Grande Valley, Edinburg, TX 78541, USA

**Keywords:** Tuberculosis, diagnosis, culture, color, DST, phenotypic, time-to-detection, contamination

## Abstract

**Background:**

With >10 million new tuberculosis (TB) cases/year, a limitation to TB control is the lack of simple and accurate tests for TB diagnosis and drug-susceptibility testing (DST) in endemic regions. We evaluated the accuracy of the first-generation, low-complexity phenotypic TB test (1G test), designed for simultaneous *Mtb* detection and resistance to isoniazid, rifampicin and moxifloxacin, suitable for resource-limited settings.

**Methods:**

A cross-sectional study was conducted using sputa from 426 possible pulmonary TB subjects from two small Mexican cities bordering Texas. The 1G test was compared against phenotypic TB detection tests in the region [acid fast bacilli smear microscopy and Mycobacteria Growth Indicator Tube (MGIT) culture], and to MGIT-DST for resistance to isoniazid, rifampicin and moxifloxacin.

**Findings:**

The 1G test demonstrated ≥98% sensitivity for *Mtb* detection, 100% sensitivity and 91% (rifampicin), 94% (isoniazid) and 97% (moxifloxacin) specificity for DST, and less contamination than the MGIT (3.5% *vs.* 8.1%; p<0.05). The 1G test time to detection (TTD) of *Mtb* and simultaneous DST was 17-days, while the MGIT-DST required two steps: 7 days for *Mtb* detection plus 14 more (total 21 days) for DST. Our study site DR-TB prevalence was 14% when testing all consecutively-enrolled participants *vs*. 6% by passive reporting.

**Interpretation:**

The 1G test is a low-complexity phenotypic TB diagnostic method that is a practical replacement to current culture-based tests. Future studies are warranted to evaluate the implementation of the 1G test in decentralized clinics that lack molecular tools, resources and expertise.

## Brief summary-clinical

The 1G test, a low-complexity phenotypic assay, detected *Mycobacterium tuberculosis* and drug resistance with high accuracy, low contamination and within 21 days, offering an affordable option for decentralized TB diagnosis and drug susceptibility testing, where molecular tools are not subsidized.

## INTRODUCTION

Tuberculosis (TB) remains one of the most prevalent infectious diseases worldwide, with an estimated 10.8 million new cases and 1·25 million deaths in 2023.^1^ TB is a major global health challenge despite advances in diagnostics, anti-mycobacterial treatment regimens, and public health interventions.^2^ This is most notable in low- and middle-income countries, where limited healthcare access and socioeconomic disparities hinder effective disease control.^2^

A obstacle to TB prevention and care is the increasing burden of drug-resistant TB (DR-TB).^2,3^ Multidrug-resistant TB (MDR-TB) is resistant to rifampicin (RIF) and isoniazid (INH), pre-extensively DR TB (pre-XDR-TB) has additional resistance to fluoroquinolone, and extensively drug-resistant TB (XDR-TB) has additional resistance to fluroquinolone plus either bedaquiline or linezolid.^3^ Factors contributing to DR-TB include poor treatment adherence and unregulated use of anti-TB drugs.^4^ Furthermore, traditional diagnostic methods such as acid-fast bacilli (AFB) smear microscopy lack sensitivity.^5^ Culture-based techniques like Löwenstein-Jensen (LJ) and BACTEC Mycobacteria Growth Indicator Tube (MGIT; Becton Dickinson, Sparks, MD) are standards for TB detection and drug susceptibility testing (DST) but are time-consuming and resource intensive.^6^ These diagnostic gaps contribute to treatment delays and amplify transmission of DR-TB. Molecular diagnostics have significantly reduced times to TB diagnosis and enabled detection of resistance mechanisms.^7^ However, high costs, infrastructure requirements, and specialized personnel needs have restricted their adoption in medium-burden and low-resource settings.^7^ Therefore, there is an urgent need for simple, rapid, and affordable diagnostic tools that improve detection of *Mtb* and its DST.

These challenges to TB control are highly relevant in medium or small cities or rural areas where most patients are treated empirically for drug-susceptible (DS)-TB, with DST reserved to high-risk groups. In Mexico, decentralized outpatient TB clinics typically establish a TB diagnosis based on clinical presentation, positive sputum smear microscopy for acid-fast bacilli, and when available, chest radiographs. When DR-TB is suspected, sputum samples are referred to Mexico City for DST, resulting in delays that extend for months. Xpert MTB/RIF (Cepheid) is available for selected cases. These limitations hinder timely treatment, promote community transmission and increase mortality. For example, in the northern sanitary jurisdictions of Tamaulipas, Mexico, the incidence of TB is at least 3-fold higher than in adjacent Texan counties in the US (35 cases/100,000 *vs.* 11 cases/100,000 in 2022, respectively).^14,15^

The first-generation (1G) phenotypic test (also known as Color-test, CX-text, TB-CX) is a low-cost, quadrant-based, thin-layer agar plate culture assay for *Mtb* detection and DST to three drugs of choice.^8^ The culture media favors accelerated *Mtb* growth detected by red colonies. Equipment requirement is minimal, making it suitable for use in TB endemic regions, including rural areas. In pilot field studies in Ethiopia, Malawi and Mozambique, we and others have shown that the 1G test detected DR-*Mtb* in sputa in a median of 14 days, with >97% agreement with LJ culture, LJ-DST for INH and RIF, or Xpert-MTB/RIF (Cepheid, Sunnyvale, CA).^8–10^ However, the performance of the 1G test from sputum has not been compared to the MGIT and MGIT-DST, which are the most sensitive and fastest phenotypic methods, although restricted by high contamination, equipment requirements, and a complex 2-step protocol for DST.^11^ Here, we evaluated whether the 1G test could be an accurate and practical alternative to the MGIT with DST, using sputa of possible TB patients from small Mexican cities bordering Texas.

## METHODS

### Study design, participant enrollment and characterization

In this cross-sectional diagnostic and DST accuracy study, we enrolled adults with possible pulmonary TB based on clinical findings (productive cough >2 weeks, weight loss, fever/chills, abnormal chest x-rays) and within 7 days of TB treatment. Sociodemographics and medical information was recorded.^12^ Diabetes was defined as fasting glucose ≥ 126 mg/dL, random ≥ 200 mg/dL or HbA1c ≥ 6·5%. HIV was determined by positive serology. The study received ethical approval from the Institutional Review Boards in Mexico (003/2022/CEI; 004/2023/CEI) and UTHealth Houston (IRB# HSC-SPH-23–0154 and HSC-SPH-12–0037).

### Sputum collection, processing and storage

**Figure 1:**
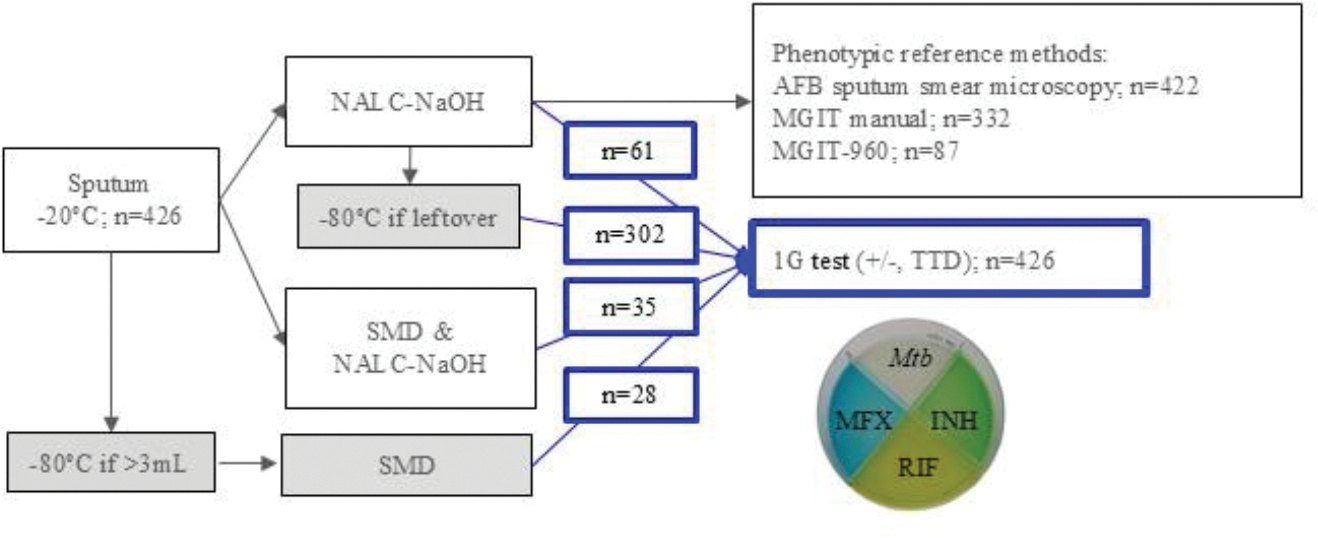
*Mtb* detection from sputum using the 1G test and conventional sputum AFB smear and MGIT culture protocols. Sputa from 426 participants with possible TB were stored at −20°C prior to batch processing within 10 days of collection (1x freeze). The number of sputa is indicated for each step, including processing for AFB smear microscopy and phenotypic tests for *Mtb* complex detection as described in the methods. Format: Gray-shaded boxes = Sputa undergoing two freezing cycles prior to thawing for the 1G test evaluation (first at −20°C upon arrival from the field, and then at −80°C with or without prior NALC-NaOH decontamination). Blue boxes = 426 sputa analyzed by the 1G test. Abbreviations: SMD = salt-mix decontaminant; TTD = time to detection of *Mtb* growth; AFB = acid-fast bacilli; MGIT manual = mycobacterium growth incubator tube manually performed; MGIT-960, automated MGIT-960 - Becton Dickinson instrument; NALC-NaOH= *N*-Acetyl-L-Cysteine - Sodium hydroxide sputum digestion and decontamination method; the 1G test, 1 ^st^ generation test.

For each patient, the same sputum was evaluated by the 1G and conventional methods. Samples were refrigerated at 4°C in the TB clinics and transported weekly to the UTHealth laboratory in Texas. Sputa were immediately stored at −20°C and thawed in the BSL-3 laboratory for aliquoting (if > 3mls) and batch processing within ten days (1x freezing). Sputa underwent standard digestion and decontamination (NALC-NaOH, Hardy Diagnostics, Santa Maria, CA).^13^ For a nested sub-analysis, some specimens were processed using an alternative salt-mix decontamination (SMD) method.^10^ Namely, sputum was mixed with the SMD preparation (1:2 v/v), vortexed briefly and incubated at room temperature for 10 min to 1 h. Leftover raw or processed sputa was stored at −80°C. Some frozen aliquots were thawed (2x freezing) for 1G testing (Fig 1 and Supplement 1).

### 1G test for mycobacterial detection and DST

The 1G test consists of a four quadrant petri dish containing an enriched and highly selective 7H11 medium that favors *Mtb* growth while deterring contamination: one quadrant for *Mtb* detection and the other three supplemented with INH (0·2 μg/mL), RIF (1·0 μg/mL), and MFX (0·25 μg/mL) for DST.^8–10,14^ Quadrants were inoculated with 100 μL of decontaminated sputum and incubated at 37°C with 5% CO_2_. *Mtb* growth was inspected for up to 42 days. Growth was evaluated by readers blinded to conventional methods’ results. Colonies displaying cording and cauliflower-like morphology under a magnifying glass, and positive for MPT-64, were classified as *Mtb*-positive.

### Phenotypic conventional methods for *Mtb* detection and DST

Concentrated sputa were used for AFB smear microscopy (Acid-Fast Stain Kit, Hardy Diagnostics). For conventional mycobacterial cultures, NALC-NAOH sputum concentrates were inoculated into MGIT media (Becton Dickinson, Franklin Lakes, NJ). A ‘MGIT-manual’ protocol was conducted between June 2020 and September 2023, with sputa cultured into 4-ml MGIT cultures and *Mtb* detected by AFB smears or colonies after sub-culture into LJ slants (details in Supplement 2). An automated MGIT-960 system was available as of October 2023 with interpretation as follows: ‘Contaminated’ if growth detected within 2 days, ‘*Mtb* positive’ if detected between 3 to 42 days and confirmed with AFB smear microscopy, and ‘negative’ if no growth was observed.^15^

Sputa with positive growth by any culture method were evaluated for the presence of AFB by smear microscopy, and if positive, evaluated for MPT-64 Ag detection (SD Bioline TB Ag MPT64, South Korea) for *Mtb* complex confirmation.^16^ Mycobacteria isolated from culture that were positive for AFB smear microscopy but negative for MPT-64 Ag were presumed to be non-tuberculous mycobacteria and excluded from analysis given their low frequency (13/439; 2·9%), and to focus on *Mtb* detection and DST (Fig S1).

The MGIT-960 DST was the gold-standard to evaluate the drug resistance of *Mtb* isolates obtained from sputum cultures positive for manual MGIT or MGIT-960. If cultures were negative or contaminated, the isolate from the control well in the 1G test was used instead. The MGIT-960 DST was performed using a commercial kit (SIRE, BD Bactec) for INH and RIF DR testing, and an in-house method for MFX at a critical concentration of 0·25 μg/mL (Thermo-Scientific Chemicals, AC457960010).^17^ Protocols were validated using reference *Mtb* clinical isolates (NIH/NIAID BEI Resources; Table S1).

### Statistical analysis

Statistical analyses were performed using SAS software vr. 9·4 (SAS Institute Inc., Cary, NC, USA). Descriptive statistics is provided. Agreement between the 1G test and conventional DST was assessed using Cohen’s kappa coefficient, with values from 0·61 to 0·80 indicating substantial agreement and 0·81 to 1·00 almost perfect agreement.^18^ Median differences were established by the Wilcoxon rank-sum test. Categorical variables were compared using the chi-square test or Fisher’s exact test when cell counts were less than five. The identification of patient characteristics independently associated with DR-TB was conducted using a logistic regression model after adjusting for age and sex and considering any variable with a p value lower than 0·2 by univariable analysis. A p-value < 0·05 was considered statistically significant. The sample size for evaluating the *Mtb* detection sensitivity of the 1G test was estimated by the McNemar test.^19^ Per WHO guidelines for low-complexity tests, we expected the 1G test sensitivity should be > 90%, or optimally above 95%.^20^ Hence, for a sensitivity within 1 to 4% of the MGIT-960, we estimated requiring 204 to 616 specimens, respectively, to achieve 80% power at a two-sided 95% confidence level.

#### Role of the funding source.

The funding source did not play a role in design, collection, analysis, interpretation, writing or decision to publish.

## RESULTS

### Participant characteristics

We evaluated the sputum from 426 participants with possible TB (Table S2). All self-identified as white Hispanics with a median age of 43 years (IQR 26) and two-thirds were males (n=294, 69%). Comorbidities included type 2 diabetes (n=188, 44%), self-reported macrovascular disease (n=92, 22%), and HIV seropositivity (n=25, 6%). Social risk factors for TB included excessive alcohol use (n=73, 17%) and frequent recreational drug use (n=87, 21%). Seventy (16%) reported a previous TB episode.

### Sputum decontamination protocols

In a sub-analysis aimed at gaining insights as to how the SMD sputum treatment performs when compared to the conventional NALC-NaOH method, we initially compared both sputum decontamination methods followed by *Mtb* growth detection in the 1G test (Fig 1 and details in Supplement 1). Among 35 sputa processed in parallel with NALC-NaOH or SMD, 34 were positive for *Mtb* growth (100% concordance). The median TTD for *Mtb* growth was similar between both sputum decontamination protocols (Table 1 using 1x frozen/defrost cycle specimens): 14·5 days for NALC-NaOH *vs.* 14 days for SMD. Given the comparable performance between both sputum processing protocols, comparison of *Mtb* growth detection for all 426 specimens in the 1G test *vs.* conventional methods were analyzed jointly, regardless of the processing method.

### Sensitivity of the 1G test for *Mtb* detection *vs*. Conventional methods

Out of the 426 sputa analyzed, the 1G test was positive for *Mtb* detection in 377 (88·5%; Fig. 1 and Fig S1). The 1G test sensitivity was compared to the detection of *Mtb* with AFB sputum smear microscopy (tested in n=422), the MGIT-manual (tested in n=332) and the automated MGIT-960 (tested in n=87) (Fig 1). Results are shown in Table 2. The sensitivity of the 1G test *vs*. positive AFB smear microscopy was 99·7% (345/346 AFB+); against positive MGIT-manual cultures was 99·6% (276/277 MGIT-manual+); against positive automated MGIT-960 cultures was 98·6% (70/71 automated MIGT-960+); and against both MGIT methods combined was 99·4% (346/348 MIGT-manual+ plus automated MGIT-960+). Altogether, the 1G test detected *Mtb* in 29 AFB smear microscopy negative specimens, 18 negative cultures, six contaminated MGIT cultures (three MGIT manual and three MGIT-960; Table 2). When comparing the 1G test to a composite of conventional tests positive by either sputum smear microscopy or manual/automated MGIT cultures, the sensitivity was maintained at 98·9%. Eight sputum specimens were positive by the 1G test but negative by all other methods. The contamination rate of the 1G test was similar to the MGIT-manual (1·2% for both tests although in different sputa), but lower than the MGIT-960 (3·5% *vs*. 8·1%; p = 0·016; Table 2).

### Accuracy of the 1G test for *Mtb* DST

The 1G test yielded simultaneous DST for RIF and INH for 376 of the 377 *Mtb*-positive sputa (one contaminated in the drug-containing well; Fig S1). MFX DST was tested in 310 sputa. Of the 376 sputa, 52 had *Mtb* resistant to at least one drug: 41 to INH, 16 to RIF, and 12 to MFX (Table 3). To evaluate the accuracy of the 1G DST when compared to the MGIT-DST as reference, 51 available DR-*Mtb* isolates were sub-cultured into MGIT media until automated growth detection. Three cultures were contaminated, leaving 49 *Mtb* isolates for analysis. Given the time resource-intensive nature of the MGIT-DST protocol, a subset of 19 DS-*Mtb* by the 1G test were also evaluated by the MGIT-DST as controls with group-matching by enrollment year and field site to the DR isolates. The performance of the 1G test relative to the MGIT-DST is shown in Table 3. The 19 DS-*Mtb* isolates from the 1G test were confirmed to be also susceptible by MGIT-DST. The 1G test suggested a higher number of DR-*Mtb* isolates *vs.* the reference MGIT-DST: 38 for INH (*vs.* 36), 15 for RIF (*vs.* 10), and 11 for MFX (*vs.* 10). Hence, the 1G test had a sensitivity of 100% for DR-*Mtb* detection to the three antibiotics, and specificity was 94% for INH, 91% for RIF, and 97% for MFX compared to MGIT-DST (Table 3). The concordance was substantial for resistance to any drug (kappa 0·86) or RIF (kappa 0·76), and almost perfect for INH and MFX (kappa 0·94 and 0·97, respectively).

### TTD for Mtb detection and DST using the 1G test vs. Conventional methods

For the 1G test, the median time for simultaneous detection of *Mtb* growth and DST was 14 days for specimens kept at −20°C prior to culture (1x freezing), and 16 to 18 days for sputa with an additional freeze-thawing cycle (2x freezing; Table 1). The median (IQR) TTD for all the 1G tests was 17 (7) days (Table 2). All the conventional methods were done with sputum frozen once at −20°C, with TTD shown in Table 2. Namely, the direct AFB sputum smear microscopy took 2 days. The MGIT-manual took 14 days for initial assessment of mycobacterial growth by AFB smear microscopy, plus an additional culture into LJ slants to confirm *Mtb* growth, resulting in a total turnaround time of approximately 42 days for detection, without DST results. The automated MGIT-960 required 7 ± 3 days for *Mtb* detection, and the additional DST required dilutions and subcultures into separate anti-TB drug-containing tubes, followed by incubation for 12–14 days for automated MGIT-960 system. Altogether this 2-step MGIT-DST protocol took 19–21 days from the time of initial sputum culture.

### Characteristics of participants with DR-TB in Mexican cities across the Texas border

The DST results were used to characterize the epidemiology patterns of DR-TB in our study population. We used the DST data from the 1G test given: i) the high concordance between the 1G test and the automated MGIT-960 (Table 3), and ii) the availability of DST data for all *Mtb*-positive 1G tests *vs*. only a subset of DS-*Mtb* (n=19) assessed by MGIT-DST. The DR-TB profiles are shown in Table 4. The prevalence of any DR-TB was 14% (52/376), with INH-R at 11%, RIF-R or MFX-R at 4%. Mono-DR was 7% for INH, 0.3% for RIF and 3% for MFX; MDR-TB was 3% and pre-XDR (MDR plus MFX-R) was 0.6%. Host characteristics were not associated with DR-TB (Table S3).

## DISCUSSION

We evaluated the 1G test in TB clinics from small cities in the Mexican border with Texas, where DR-TB testing in not locally available, and referrals to central clinics are limited to cases with high risk of DR-TB. We found that the 1G test was: i) more sensitive for *Mtb* detection than the MGIT manual (18 positive specimens by 1G but negative by MGIT manual), but comparable to automated MGIT cultures, and ii) less prone to contamination. iii) The 1G test had a good concordance with the MGIT-DST for INH, RIF and MFX, with the added advantage of less contamination, faster results and a simpler protocol; iv) the 1G test had similar TTD for *Mtb* but shorter for DST *vs.* automated MGIT-DST; and v) the 1G test comprised a simple one-step process compared to additional supplies, personnel and equipment for the MGIT-DST. Altogether, the 1G test is a low-complexity phenotypic TB diagnostics that offers a practical alternative to phenotypic tests for *Mtb* detection and DST (e.g. LJ or MGIT with DST). Its simplicity makes it suitable for use in decentralized laboratories in mid- and high-burden TB regions, where smear microscopy is routine, biosafety cabinets are available and molecular testing is not feasible due to high cost and limited resources (e.g. trained personnel, expensive instrumentation). Additionally, while molecular testing targets mutations conferring resistance, there remain DR-TB cases that can only be detected using phenotypic methods.

Our study setting shares the limitations for TB diagnosis as many other regions worldwide. Namely, Mexican TB referral clinics on the border with Texas do not offer routine testing for DR-TB, unless the individual is younger than five, has failed treatment, is immunocompromised or poses a high risk for DR-TB.^21^ In some high TB burden countries, Xpert (Cepheid) is replacing AFB smear microscopy testing, but in Mexico this technology is not subsidized, is costly (>$50/cartridge) and not readily available.^7^ Instead, DST is centralized and results can take up to 6 months.^7^ Our results provide support for the value of the 1G test as a technically simple, reliable phenotypic method for diagnosis of TB and DR-TB in these types of settings, and without need for additional infrastructure or biosafety considerations.

The 1G test had comparable performance when sputa were processed by the NALC-NaOH or the SMD method. The use of SMD for digestion and decontamination plus the 1G test for *Mtb* isolation and DST testing, has the advantage of requiring less sputum (150 μL), and can be performed without equipment. Namely, the SMD vortexing step can be replaced by handshaking or mixing using a disposable transfer pipet to avoid aerosol or froth generation.^10,14^ Furthermore, the 37°C incubation can be done without CO_2_ or even at room temperature in countries where ambient temperature is closer to 37°C, although with a longer TTD (Torrelles, unpublished findings). However, further evidence is needed to determine the biosafety implications of these modifications, including whether personnel safety still requires the use of a biosafety cabinet.

The 1G test had a shorter 2-week turnaround time, and a simpler and economical 1-step protocol *vs*. the 2-step MGIT and MGIT-DST protocols that require equipment and additional steps. The TTD of the 1G test was faster *vs*. the standard 28 to 42 days for *Mtb* detection with LJ cultures, and prior to sub-cultures for DST, with even shorter TTD of 10 to 13 days for the 1G test in other settings using fresh sputa.^8^

Despite good agreement between the 1G test and MGIT for DST, the nature of their discordant results deserve further evaluation. RIF showed the highest discrepancy. This may reflect RIF instability during the 42-day 1G incubation or inability of the MGIT-DST to detect low-level RIF-resistance in some strains, considering the recent WHO recommendation to lower the MGIT-DST RIF critical concentration from 1·0 to 0·5 mg/L.^22–26^ This lower concentration is already used in the 1G test. Understanding the molecular basis for discrepancies is clinically relevant given associations between low-level RIF-resistance and poor treatment outcomes.^26^ Genotyping is in progress to clarify if discordant findings represent false positive results.

DR-TB was estimated at 6% in northern Tamaulipas in 2020 by passive reporting, while our results between 2020 and 2024 suggested that DR-TB is more than twice as high (14%). The passive underestimation is likely due to the lack of routine DST testing, although sampling bias in our study cannot be excluded. The MDR-TB rate of 3% in our study population is comparable to 3·2% globally.^1^ The 4% prevalence of RIF resistance using the 1G test was below the global estimate of 6·9%, and was even lower when determined by the MGIT-DST.^27^ Only two of 14 (14%) MDR-TB cases had additional resistance to MFX (pre-XDR TB), which is lower than the reported WHO rate of 20%.^1^

Detection of MFX resistant isolates was unexpected as this drug has not been introduced for TB treatment locally. A possible explanation is cross-resistance due to the unprescribed use of fluoroquinolones in our study population.^28^ The WHO recommends MFX for treating DS-TB, INH mono-DR TB, and MDR-TB, but our finding points the need for MFX DST prior to its use in Mexican border TB patients.^29^ Unlike previous studies, we found no association between DR-TB and host factors^30^ which may reflect the small sample number of DR-TB cases in our study.

A study limitation is the lack of simultaneous testing with the 1G test and conventional methods, which required an additional freezing step for two-thirds of the samples tested with the 1G test. Despite this disadvantage, the 1G test demonstrated a robust performance except for a median three-day delay in TTD. Even though there was perfect concordance between the 1G test and MGIT-DST for a subset of pan-sensitive *Mtb* isolates identified by the 1G test, we cannot rule out missing DR-*Mtb* isolates unique to the MGIT-DST. The RIF-R prevalence in our community based on the 1G test should be interpreted with caution given its higher prevalence *vs*. MGIT-DST. Most study participants had a positive AFB sputum smear microscopy and our conclusions should take into consideration this potential bias.

In conclusion, our findings build upon previous work to provide support for the 1G test as an accurate, simple and affordable alternative to current phenotypic methods for TB detection in resource-limited high-burden settings. Used in parallel with sputum smear microscopy at centralized or decentralized clinics, it can enhance mycobacterial detection sensitivity and enable DST, especially where molecular tools like GeneXpert are not subsidized. Future studies are warranted to evaluate the implementation of the 1G test in decentralized clinics lacking molecular diagnostic capacity, with simple modifications as needed, such as the use of SMD, transfer pipets for mixing with minimal aerosol generation, and incubations at room temperature.

## Supplementary Material

Supplementary Material

## Figures and Tables

**Table 1.** Time-to-detection of *Mtb* growth using the 1G test, by sputum processing and freezing events

Sputum processing and freezing events	n positive^1^	TTD in days (median ± IQR)	Range	P value

NALC-NaOH, Frozen 1x ^2^	86	14 ± 6	4–42 days	<0·0001
NALC-NaOH, Frozen 2x	265	18 ± 5	4–42 days	

SMD, Frozen 1x ^2^	34	14 ± 5	4–42 days	0·02
SMD, Frozen 2x	26	16 ± 7	7–42 days	

1 Only data from the 377 sputum specimens that were positive for *Mtb* growth on the 1G test are shown.

2 The results of 34 sputum specimens that were frozen once are shown twice given the parallel processing using NALC-NaOH and SMD. Abbreviations: SMD, Salt-mix decontamination; TTD, Time to Detection of *Mtb* growth; 1x, Sputum frozen once at −20°C prior to weekly processing and culture; 2x, Sputum frozen twice: first at −20°C and leftover re-frozen at −80°C prior to seeding in the 1G test plate.

**Table 2.** Performance of the 1G test and phenotypic reference standard methods for *Mtb* detection from sputum ^1^

	Reference method			1G test ^2^	Other performance statistics		
	*Mtb*+	*Mtb*-	Cont	Sensitivity (95% CI)	Test	Cont n (%)	TTD in days Median ± IQR ^3^
		
**1G test *vs*. sputum AFB (n=422)**							
*Mtb*+	345	29	NA	99·7%	1G	7 (1·7%)	17 ± 7
*Mtb*-	1	40	NA	(98%, 100%)	AFB	NA	2 days
Contamination	2	5	NA				

**1G test *vs*. MGIT-manual (n=332)**							
Mtb+	276	18	3	99·6%	1G	4 (1·2%)	17 ± 7
Mtb-	1	29	1	(98%, 100%)	MGIT-M	4 (1·2%)	>14 days^4^
Contamination	1	3	0				

**1G test *vs*. MGIT-960 (n=87)**							
Mtb+	70	0	3	98·6%	1G	3 (3·5%)4	17 ± 10
Mtb-	1	8	2	(93%, 100%)	MGIT-960	7 (8·1%)	7 ± 3
Contamination	1	0	2				

**1G test vs. all MGIT (n=419)**							
Mtb+	346	18	6	99·4%	1G	7 (1·7%)	17 ± 7
Mtb-	2	37	3	(98%, 100%)	MGIT	11 (2·6%)	N/A
Contamination	2	3	2				

**1G test *vs*. Composite (n=426)**							
Mtb+	369	8	NA	98·9%	1G	7 (1·6%)	17 ± 7
Mtb-	4	38	NA	(97%, 100%)	Composite	N/A	N/A
Contamination	2	5	N/A				

1 Total number of sputum specimens tested by the 1G test and the listed method(s).

2 Calculations exclude the contaminated results.

3 The median (IQR) TTD for the 1G test with specimens frozen 1x is 14 (6), which are conditions comparable to the reference methods, but data showing 17 days is due to inclusion of specimens frozen 2x as shown in Table 3.

4 p value = 0·016. Abbreviations: Cont, contamination; TTD, Time to detection of *Mtb* growth (for the 1G this is also the time for DST); IQR, interquartile range, MGIT, Mycobacterial growth indicator tube.

**Table 3.** Performance of the 1G test for detection of DR-*Mtb* against MGIT-DST ^1^

1G test results	MGIT results (n)			1G test vs MGIT ^2^		DST concordance ^2^	
	DR	DS	Cont ^2^	Sensitivity (95% CI)	Specificity (95% CI)	Kappa (95% CI)	Kappa interpretation

**Any DR**							
1G test - DR	45	4	3	100%	83%	0·86	Substantial
1G test - DS	0	19	0	(92–100%)	(61–94%)	(0·73–0·99)	agreement

**INH**							
1G test - DR	36	2	3	100%	94%	0·94	Almost perfect
1G test - DS	0	30	0	(90–100%)	(78–99%)	(0·87–1·00)	agreement

**RIF**							
1G test - DR	10	5	1	100%	91%	0·76	Substantial
1G test - DS	0	53	2	(69–100%)	(80–98%)	(0·56–0·96)	agreement

**MFX**							
1G test - DR	10	1	1	100%	97%	0·97	Almost perfect
1G test - DS	0	30	2	(70–100%)	(84–100%)	(0·83–1·00)	agreement

1 DST conducted in all the isolates with DR-Mtb (n=52) plus 19 group-matched DS-Mtb per the 1G test;

2 Contaminated results were excluded from the sensitivity, specificity or concordance analysis. Abbreviations: DR=drug resistant; DS=drug susceptible; Cont=contaminated; Kappa=concordance ; CI, confidence intervals.

**Table 4.** Prevalence of DR-TB in pulmonary TB patients from Mexican border communities ^1^

Type of DR	n with DR	Total DR (%) in all *Mtb* ^2^	Total DR (%) in any DR ^2^
**Any DR**			
Any INH-R	41	11%	79%
Any RIF-R	16	4%	31%
Any MFX-R	12	4%	23%
**Mono-DR**			
Mono INH-R	27	7%	52%
Mono RIF-R	1	0·3%	2%
Mono MFX-R	9	3%	17%
**Multiple-DR**			
INH/RIF (MDR)	12	3%	23%
INH/RIF/MFX (Pre-XDR)	2	0·6%	4%
RIF/MFX	1	0·3%	2%

Total with any DR	52	14%	100%

1 Prevalence based on DR-*Mtb* detected by the 1G test;

2 Denominator is 376 for RIF- and INH-DR, and 310 for analysis containing MFX-DR. Abbreviations: INH, isoniazid; RIF, rifampicin; MFX, moxifloxacin; R, resistant; MDR, multi-drug resistant; XDR, extreme drug resistant; DR, drug resistant.

## Data Availability

Data collected for this study includes individual participant data and a data dictionary. The datasets used during the current study are available from the corresponding author on reasonable request.
