# Accuracy of the Phenotypic 1G Test to Detect *Mycobacterium tuberculosis* and Drug Resistance From Sputa in the US-Mexico Border

**DOI:** 10.1093/infdis/jiaf638

**Published:** 2025-12-23

**Authors:** Mia Aguirre, Doris Ayala, Juan Ignacio Garcia, Yoscelina E Martinez-Lopez, Amberlee D Hicks, Nadine Chacon, Ashley Gay-Cobb, Alyssa Schami, Selena Zavala-Perez, Ilse A Dominguez-Trejo, America M Cruz-Gonzalez, Raul Loera-Salazar, Javier E Rodríguez-Herrera, Esperanza M Garcia-Oropesa, Miryoung Lee, Adrian Rendón, Shu-Hua Wang, Marcel Yotebieng, Carlton A Evans, Jordi B Torrelles, Blanca I Restrepo

**Affiliations:** Department of Epidemiology, School of Public Health, University of Texas Health Science Center at Houston, Brownsville; Department of Epidemiology, School of Public Health, University of Texas Health Science Center at Houston, Brownsville; Population Health and Host Pathogens Interactions Programs; International Center for the Advancement of Research and Education, Texas Biomedical Research Institute, San Antonio; Department of Epidemiology, School of Public Health, University of Texas Health Science Center at Houston, Brownsville; Population Health and Host Pathogens Interactions Programs; International Center for the Advancement of Research and Education, Texas Biomedical Research Institute, San Antonio; Population Health and Host Pathogens Interactions Programs; Population Health and Host Pathogens Interactions Programs; International Center for the Advancement of Research and Education, Texas Biomedical Research Institute, San Antonio; Population Health and Host Pathogens Interactions Programs; International Center for the Advancement of Research and Education, Texas Biomedical Research Institute, San Antonio; Department of Epidemiology, School of Public Health, University of Texas Health Science Center at Houston, Brownsville; Department of Epidemiology, School of Public Health, University of Texas Health Science Center at Houston, Brownsville; Departamento Estatal de Micobacteriosis, Secretaría de Salud de Tamaulipas; Departamento Estatal de Micobacteriosis, Secretaría de Salud de Tamaulipas; Departamento Estatal de Micobacteriosis, Secretaría de Salud de Tamaulipas; Unidad Académica Multidisciplinaria Reynosa-Aztlán, Universidad Autónoma de Tamaulipas, Reynosa; Department of Epidemiology, School of Public Health, University of Texas Health Science Center at Houston, Brownsville; Centro de Investigación, Prevención y Tratamiento de Infecciones Respiratorias and Hospital Universitario “Dr Jose Eleuterio Gonzalez,” Nuevo Leon, México; International Center for the Advancement of Research and Education, Texas Biomedical Research Institute, San Antonio; Division of Infectious Disease, Department of Internal Medicine, College of Medicine, The Ohio State University, Columbus; International Center for the Advancement of Research and Education, Texas Biomedical Research Institute, San Antonio; Division of General Internal Medicine, Department of Medicine Albert Einstein College of Medicine, The Bronx, New York; Innovation for Health and Development, Section of Adult Infectious Disease, Department of Infectious Disease, Imperial College London, South Kensington Campus, United Kingdom; Inovacion Por la Salud Y el Desarrollo, Asociacion Benefica PRISMA; Innovation for Health and Development, Laboratory of Research and Development, Faculty of Sciences and Engineering, Universidad Peruana Cayetano Heredia, Lima, Peru; Population Health and Host Pathogens Interactions Programs; International Center for the Advancement of Research and Education, Texas Biomedical Research Institute, San Antonio; Department of Epidemiology, School of Public Health, University of Texas Health Science Center at Houston, Brownsville; International Center for the Advancement of Research and Education, Texas Biomedical Research Institute, San Antonio; School of Medicine, South Texas Diabetes and Obesity Institute and Department of Human Genetics, University of Texas Rio Grande Valley, Edinburg

**Keywords:** culture, diagnosis, DST, phenotypic, tuberculosis

## Abstract

**Background:**

With >10 million new tuberculosis (TB) cases per year, a limitation to TB control is the lack of simple and accurate tests for diagnosis and drug susceptibility testing (DST) in endemic regions. We evaluated the accuracy of the first-generation, low-complexity phenotypic TB test (1G test), designed for simultaneous *Mycobacterium tuberculosis* (*Mtb*) detection and resistance to isoniazid, rifampicin, and moxifloxacin, suitable for resource-limited settings.

**Methods:**

A cross-sectional study was conducted with sputa from 426 possible pulmonary TB cases from 2 small Mexican cities bordering Texas. The 1G test was compared against phenotypic TB detection tests in the region, specifically acid-fast bacilli smear microscopy and Mycobacteria Growth Indicator Tube (MGIT) culture, as well as MGIT-DST for resistance to isoniazid, rifampicin, and moxifloxacin.

**Results:**

The 1G test demonstrated ≥98% sensitivity for *Mtb* detection; 100% sensitivity and 91% (rifampicin), 94% (isoniazid), and 97% (moxifloxacin) specificity for DST; and less contamination than the MGIT (3.5% vs 8.1%, *P* < .05). The 1G test time to detection of *Mtb* and simultaneous DST was 17 days, while the MGIT-DST required 2 steps: 7 days for *Mtb* detection plus 14 more days for DST (21 days total). Our study site drug-resistant TB prevalence was 14% when testing all consecutively enrolled participants vs 6% by passive reporting.

**Conclusions:**

The 1G test is a low-complexity phenotypic TB diagnostic method that is a practical replacement to current culture-based tests. Future studies are warranted to evaluate implementation of the 1G test in decentralized clinics that lack molecular tools, resources, and expertise.

## Teaser

The 1G test, a low-complexity phenotypic assay, detected *Mycobacterium tuberculosis* and drug resistance with high specificity, low contamination, and short turnaround (<21 days), offering an affordable option for decentralized tuberculosis diagnosis and drug susceptibility testing.

Tuberculosis (TB) remains one of the most prevalent infectious diseases worldwide, with an estimated 10.8 million new cases and 1.25 million deaths in 2023 [1]. TB is a major global health challenge despite advances in diagnostics, antimycobacterial treatment regimens, and public health interventions [2]. This is most notable in low- and middle-income countries, where limited health care access and socioeconomic disparities hinder effective disease control [2].

A obstacle to TB prevention and care is the increasing burden of drug-resistant TB (DR-TB) [2, 3]. Multidrug-resistant TB (MDR-TB) is resistant to rifampicin (RIF) and isoniazid (INH); pre-extensively DR-TB has additional resistance to fluoroquinolone; and extensively DR-TB has additional resistance to fluroquinolone plus either bedaquiline or linezolid [3]. Factors contributing to DR-TB include poor treatment adherence and unregulated use of anti-TB drugs [4]. Furthermore, traditional diagnostic methods such as acid-fast bacilli (AFB) smear microscopy lack sensitivity [5]. Culture-based techniques such as Löwenstein-Jensen (LJ) and BACTEC Mycobacteria Growth Indicator Tube (MGIT; Becton Dickinson) are standards for TB detection and drug susceptibility testing (DST) but are time-consuming and resource intensive [6]. These diagnostic gaps contribute to treatment delays and amplify transmission of DR-TB. Molecular diagnostics have significantly reduced times to TB diagnosis and enabled detection of resistance mechanisms [7]. However, high costs, infrastructure requirements, and specialized personnel needs have restricted their adoption in medium-burden and low-resource settings [7]. Therefore, there is an urgent need for simple, rapid, and affordable diagnostic tools that improve detection of *Mycobacterium tuberculosis* (*Mtb*) and its DST.

These challenges to TB control are highly relevant in medium or small cities or rural areas where most patients are treated empirically for drug-susceptible TB, with DST reserved to high-risk groups. In Mexico, decentralized outpatient TB clinics typically establish a TB diagnosis based on clinical presentation, positive sputum smear microscopy for AFB, and when available, chest radiographs. When DR-TB is suspected, sputum samples are referred to Mexico City for DST, resulting in delays that extend for months. Xpert MTB/RIF (Cepheid) is available for selected cases. These limitations hinder timely treatment, promote community transmission, and increase mortality. For example, in the northern sanitary jurisdictions of Tamaulipas, Mexico, the incidence of TB is at least 3-fold higher than in adjacent Texan counties in the United States (35 vs 11 cases/100 000 in 2022, respectively) [8, 9].

The first-generation (1G) phenotypic test (also known as Colour Test, CX-test, TB-CX) is a low-cost, quadrant-based, thin-layer agar plate culture assay for *Mtb* detection and DST to 3 drugs of choice [10]. The culture media favors accelerated *Mtb* growth detected by red colonies. Equipment requirement is minimal, making it suitable for use in TB endemic regions, including rural areas. In pilot field studies in Ethiopia, Malawi, and Mozambique, we and others have shown that the 1G test detected DR-*Mtb* in sputa in a median 14 days, with >97% agreement with LJ culture, LJ-DST for INH and RIF, or Xpert-MTB/RIF (Cepheid) [10–12]. However, the performance of the 1G test from sputum has not been compared with the MGIT and MGIT-DST, which are the most sensitive and fastest phenotypic methods, despite being restricted by high contamination, equipment requirements, and a complex 2-step protocol for DST [13]. Here, we evaluated whether the 1G test could be an accurate and practical alternative to the MGIT with DST, using sputa of patients with possible TB from small Mexican cities bordering Texas.

## METHODS

### Study Design, Participant Enrollment, and Characterization

In this cross-sectional diagnostic and DST accuracy study, we enrolled adults with possible pulmonary TB based on clinical findings (productive cough >2 weeks, weight loss, fever/chills, abnormal chest x-ray findings) and within 7 days of TB treatment. Sociodemographics and medical information was recorded [14]. Diabetes was defined as fasting glucose ≥126 mg/dL, random ≥200 mg/dL, or hemoglobin A_1c_ ≥6.5%. HIV was determined by positive serology. The study received ethical approval from the institutional review boards in Mexico (003/2022/CEI, 004/2023/CEI) and UTHealth Houston (HSC-SPH-23-0154, HSC-SPH-12-0037).

### Sputum Collection, Processing, and Storage

**Figure 1.**
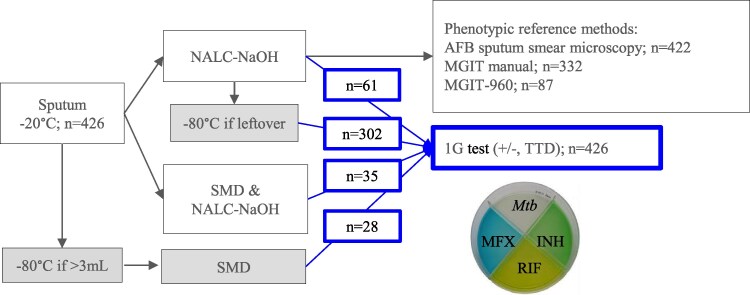
*Mtb* detection from sputum per the 1G test and conventional sputum AFB smear and MGIT culture protocols. Sputa from 426 participants with possible TB were stored at –20 °C prior to batch processing within 10 days of collection (1× freeze). The number of sputa is indicated for each step, including processing for AFB smear microscopy and phenotypic tests for *Mtb* complex detection as described in the methods. Gray-shaded boxes = sputa undergoing 2 freezing cycles prior to thawing for the 1G test evaluation: first at −20 °C upon arrival from the field, then at −80 °C with or without prior NALC-NaOH decontamination. Blue boxes = 426 sputa analyzed by the 1G test. 1G test, first-generation test. Abbreviations: AFB, acid-fast bacilli; INH, isoniazid; MFX, moxifloxacin; MGIT manual, Mycobacteria Growth Indicator Tube manually performed; MGIT-960, automated MGIT-960 (Becton Dickinson) instrument; *Mtb*, *Mycobacterium tuberculosis*; NALC-NaOH, *N*-acetyl-L-cysteine–sodium hydroxide sputum digestion and decontamination method; RIF, rifampicin; SMD, salt-mix decontamination; TTD, time to detection of *Mtb* growth.

For each patient, the same sputum was evaluated by the 1G and conventional methods. Samples were refrigerated at 4 °C in the TB clinics and transported weekly to the UTHealth laboratory in Texas. Sputa were immediately stored at −20 °C and thawed in a biosafety level 3 laboratory for aliquoting (if >3 mL) and batch processing within 10 days (1× freezing). Sputa underwent standard digestion and decontamination (NALC-NaOH; Hardy Diagnostics) [15]. For a nested subanalysis, some specimens were processed with an alternative salt-mix decontamination (SMD) method [12]. Namely, sputum was mixed with the SMD preparation (1:2 v/v), vortexed briefly, and incubated at room temperature for 10 minutes to 1 hour. Leftover raw or processed sputa were stored at −80 °C. Some frozen aliquots were thawed (2× freezing) for 1G testing (Figure 1, Supplement 1).

### 1G Test for Mycobacterial Detection and DST

The 1G test consists of a 4-quadrant Petri dish containing an enriched and highly selective 7H11 medium that favors *Mtb* growth while deterring contamination: 1 quadrant for *Mtb* detection and the other 3 supplemented with INH (0.2 µg/mL), RIF (1.0 µg/mL), and moxifloxacin (MFX; 0.25 µg/mL) for DST [8, 10–12]. Quadrants were inoculated with 100 µL of decontaminated sputum and incubated at 37 °C with 5% CO₂. *Mtb* growth was inspected for up to 42 days. Growth was evaluated by readers blinded to conventional methods’ results. Colonies displaying cording and cauliflower-like morphology under a magnifying glass and positive for MPT-64 were classified as *Mtb* positive.

### Phenotypic Conventional Methods for *Mtb* Detection and DST

Concentrated sputa were used for AFB smear microscopy (Acid-Fast Stain Kit; Hardy Diagnostics). For conventional mycobacterial cultures, NALC-NAOH sputum concentrates were inoculated into MGIT media (Becton Dickinson). A “MGIT-manual” protocol was conducted between June 2020 and September 2023, with sputa cultured into 4-mL MGIT cultures and *Mtb* detected by AFB smears or colonies after subculture into LJ slants (Supplement 2). An automated MGIT-960 system was available as of October 2023 with interpretation as follows: “contaminated” if growth detected within 2 days, “*Mtb* positive” if detected between 3 and 42 days and confirmed with AFB smear microscopy, and “negative” if no growth was observed [9].

Sputa with positive growth by any culture method were evaluated for the presence of AFB by smear microscopy and, if positive, evaluated for MPT-64 Ag detection (SD Bioline TB Ag MPT64) for *Mtb* complex confirmation [16]. Mycobacteria isolated from culture that were positive for AFB smear microscopy but negative for MPT-64 Ag were presumed to be nontuberculous mycobacteria and excluded from analysis given their low frequency (13/439, 2.9%) and to focus on *Mtb* detection and DST (Supplementary Figure 1).

MGIT-960 DST was the gold standard to evaluate the drug resistance of *Mtb* isolates obtained from sputum cultures positive for MGIT-manual or MGIT-960. If cultures were negative or contaminated, the isolate from the control well in the 1G test was used instead. MGIT-960 DST was performed with a commercial kit (SIRE; BD Bactec) for INH and RIF DR testing and an in-house method for MFX at a critical concentration of 0.25 µg/mL (AC457960010; Thermo-Scientific Chemicals) [17]. Protocols were validated by reference *Mtb* clinical isolates (BEI Resources, National Institute of Allergy and Infectious Diseases, National Institutes of Health; Supplementary Table 1).

### Statistical Analysis

Statistical analyses were performed in SAS software version 9.4 (SAS Institute Inc). Descriptive statistics are provided. Agreement between the 1G test and conventional DST was assessed by Cohen κ coefficient, with values from 0.61 to 0.80 indicating substantial agreement and 0.81 to 1.00 almost perfect agreement [18]. Median differences were established by the Wilcoxon rank sum test. Categorical variables were compared with the χ^2^ test or the Fisher exact test when cell counts were <5. The identification of patient characteristics independently associated with DR-TB was conducted in a logistic regression model after adjusting for age and sex and considering any variable with *P* < .2 by univariable analysis. *P* < .05 was considered statistically significant. The sample size for evaluating the *Mtb* detection sensitivity of the 1G test was estimated by the McNemar test [19]. Per World Health Organization (WHO) guidelines for low-complexity tests, we expected the 1G test sensitivity to be >90% or optimally >95% [20]. Hence, for a sensitivity within 1% to 4% of the MGIT-960, we estimated requiring 204 to 616 specimens, respectively, to achieve 80% power at a 2-sided 95% CI.

### Role of the Funding Source

The funding source did not play a role in the design, collection, analysis, interpretation, writing, or decision to publish.

## RESULTS

### Participant Characteristics

We evaluated the sputum from 426 participants with possible TB (Supplementary Table 2). All self-identified as White Hispanics with a median age of 43 years (IQR, 26) and two-thirds were male (n = 294, 69%). Comorbidities included type 2 diabetes (n = 188, 44%), self-reported macrovascular disease (n = 92, 22%), and HIV seropositivity (n = 25, 6%). Social risk factors for TB included excessive alcohol use (n = 73, 17%) and frequent recreational drug use (n = 87, 21%). Seventy (16%) reported a previous TB episode.

### Sputum Decontamination Protocols

In a subanalysis aimed at gaining insights into how the SMD sputum treatment performs when compared with the conventional NALC-NaOH method, we initially compared both sputum decontamination methods, followed by *Mtb* growth detection in the 1G test (Figure 1, Supplement 1). Among 35 sputa processed in parallel with NALC-NaOH or SMD, 34 were positive for *Mtb* growth (100% concordance). The median time to detection (TTD) for *Mtb* growth was similar between the sputum decontamination protocols (Table 1 with 1× frozen/defrost cycle specimens): 14.5 days for NALC-NaOH vs 14 days for SMD. Given the comparable performance between the sputum processing protocols, comparison of *Mtb* growth detection for all 426 specimens in the 1G test vs conventional methods was analyzed jointly, regardless of the processing method.

**Table 1.** TTD of *Mtb* Growth per the 1G Test by Sputum Processing and Freezing Events

Sputum Processing: Freezing Events	No. Positive^a^	TTD, d (Median ± IQR)	Range, d	*P* Value
NALC-NaOH				<.0001
Frozen 1x^b^	86	14 ± 6	4–42	
Frozen 2x	265	18 ± 5	4–42	
SMD				0.02
Frozen 1x^b^	34	14 ± 5	4–42	
Frozen 2x	26	16 ± 7	7–42	

Abbreviations: 1×, sputum frozen once at −20 °C prior to weekly processing and culture; 2×, sputum frozen twice (first at −20 °C and leftover refrozen at −80 °C prior to seeding in the 1G test plate); *Mtb*, *Mycobacterium tuberculosis*; SMD, salt-mix decontamination; TTD, time to detection of *Mtb* growth.

^a^Only data from the 377 sputum specimens that were positive for *Mtb* growth on the 1G test are shown.

^b^The results of 34 sputum specimens that were frozen once are shown twice given the parallel processing by NALC-NaOH and SMD.

### Sensitivity of the 1G Test for *Mtb* Detection vs Conventional Methods

Out of the 426 sputa analyzed, the 1G test was positive for *Mtb* detection in 377 (88.5%; Figure 1, Supplementary Figure 1). The 1G test sensitivity was compared with the detection of *Mtb* with AFB sputum smear microscopy (tested in n = 422), the MGIT-manual (tested in n = 332), and the automated MGIT-960 (tested in n = 87). Results are shown in Table 2. The sensitivity of the 1G test vs positive AFB smear microscopy was 99.7% (345/346 AFB+); against positive MGIT-manual cultures, 99.6% (276/277 MGIT-manual+); against positive automated MGIT-960 cultures, 98.6% (70/71 automated MIGT-960+); and against both MGIT methods combined, 99.4% (346/348 MIGT-manual+ plus automated MGIT-960+). Altogether, the 1G test detected *Mtb* in 29 negative specimens on AFB smear microscopy, 18 negative cultures, and 6 contaminated MGIT cultures (3 MGIT manual and 3 MGIT-960). When the 1G test was compared against a composite of conventional tests positive by either sputum smear microscopy or manual/automated MGIT cultures, the sensitivity was maintained at 98.9%. Eight sputum specimens were positive by the 1G test but negative by all other methods. The contamination rate of the 1G test was similar to the MGIT-manual (1.2% for both tests although in different sputa) but lower than the MGIT-960 (3.5% vs 8.1%, *P* = .016).

**Table 2.** Performance of the 1G Test and Phenotypic Reference Standard Methods for *Mtb* Detection From Sputum

	Reference Method			1G Test^a^	Other Performance Statistics		
	*Mtb*+	*Mtb*–	Cont	Sensitivity (95% CI), %	Test	Cont, No. (%)	TTD (d), Median ± IQR^b^
1G test vs sputum AFB (n = 422)							
* Mtb*+	345	29	…	99.7 (98–100)	1G	7 (1.7)	17 ± 7
* Mtb*–	1	40	…		AFB	…	2
Cont	2	5	…				
1G test vs MGIT-manual (n = 332)							
* Mtb*+	276	18	3	99.6 (98–100)	1G	4 (1.2)	17 ± 7
* Mtb*–	1	29	1		MGIT-M	4 (1.2)	≥14
Cont	1	3	0				
1G test vs MGIT-960 (n = 87)							
*Mtb*+	70	0	3	98.6 (93–100)	1G	3 (3.5)^c^	17 ± 10
*Mtb*–	1	8	2		MGIT-960	7 (8.1)	7 ± 3
Cont	1	0	2				
1G test vs all MGIT (n = 419)							
*Mtb*+	346	18	6	99.4 (98–100)	1G	7 (1.7)	17 ± 7
*Mtb*–	2	37	3		MGIT	11 (2.6)	…
Cont	2	3	2				
1G test vs composite (n = 426)							
*Mtb*+	369	8	…	98.9 (97–100)	1G	7 (1.6)	17 ± 7
*Mtb*–	4	38	…		Composite	…	…
Cont	2	5	…				

Table includes total number of sputum specimens tested by the 1G test and the listed methods.

Abbreviations: Cont, contamination; MGIT, Mycobacteria Growth Indicator Tube; *Mtb*, *Mycobacterium tuberculosis*; TTD, time to detection of *Mtb* growth in days (d). F for the 1G, this TTD is also the time for DST.

^a^Calculations exclude the contaminated results.

^b^The median (IQR) TTD for the 1G test with specimens frozen 1× is 14 (6), which is comparable to the reference methods, but data showing 17 days are due to inclusion of specimens frozen 2× as shown in Table 3.

^c^ *P* = .016.

### Accuracy of the 1G Test for *Mtb* DST

The 1G test yielded simultaneous DST for RIF and INH for 376 of the 377 *Mtb*-positive sputa, with 1 contaminated in the drug-containing well (Supplementary Figure 1). MFX DST was tested in 310 sputa. Of the 376 sputa, 52 had *Mtb* resistant to at least 1 drug: 41 to INH, 16 to RIF, and 12 to MFX (Table 3). To evaluate the accuracy of the 1G DST when compared with the MGIT-DST as reference, 51 available DR-*Mtb* isolates were subcultured into MGIT media until automated growth detection. Three cultures were contaminated, leaving 49 *Mtb* isolates for analysis. Given the time- and resource-intensive nature of the MGIT-DST protocol, a subset of 19 drug-susceptible *Mtb* isolates by the 1G test were also evaluated by the MGIT-DST as controls with group matching by enrollment year and field site to the DR isolates. The performance of the 1G test relative to the MGIT-DST is shown in Table 3. The 19 drug-susceptible *Mtb* isolates from the 1G test were confirmed to be susceptible by MGIT-DST. The 1G test suggested a higher number of DR-*Mtb* isolates vs the reference MGIT-DST: 38 for INH (vs 36), 15 for RIF (vs 10), and 11 for MFX (vs 10). Hence, the 1G test had a sensitivity of 100% for DR-*Mtb* detection to the 3 antibiotics, and specificity was 94% for INH, 91% for RIF, and 97% for MFX as compared with MGIT-DST. The concordance was substantial for resistance to any drug (κ = 0.86) or RIF (κ = 0.76) and almost perfect for INH and MFX (κ = 0.94 and 0.97, respectively).

**Table 3.** Performance of the 1G Test for Detection of DR-*Mtb* Against MGIT-DST

	MGIT Results, No.			1G Test vs MGIT, %		DST Concordance	
1G Test Results	DR	DS	Cont^a^	Sensitivity (95% CI)	Specificity (95% CI)	κ (95% CI)	Interpretation
Any DR				100 (92–100)	83 (61–94)	.86 (.73–.99)	Substantial agreement
DR	45	4	3				
DS	0	19	0				
INH				100 (90–100)	94 (78–99)	.94 (.87–1.00)	Almost perfect agreement
DR	36	2	3				
DS	0	30	0				
RIF				100 (69–100)	91 (80–98)	.76 (.56–.96)	Substantial agreement
DR	10	5	1				
DS	0	53	2				
MFX				100 (70–100)	97 (84–100)	.97 (.83–1.00)	Almost perfect agreement
DR	10	1	1				
DS	0	30	2				

DST conducted in all the isolates with DR-*Mtb* (n = 52) plus 19 group-matched DS-*Mtb* per the 1G test.

Abbreviations: Cont, contaminated; DR, drug resistant; DS, drug susceptible; DST, drug susceptibility testing; MGIT, Mycobacteria Growth Indicator Tube; *Mtb*, *Mycobacterium tuberculosis*.

^a^Contaminated results were excluded from the sensitivity, specificity, or concordance analysis.

### TTD for *Mtb* and DST per the 1G Test vs Conventional Methods

For the 1G test, the median time for simultaneous detection of *Mtb* growth and DST was 14 days for specimens kept at −20 °C prior to culture (1× freezing) and 16 to 18 days for sputa with an additional freeze-thawing cycle (2× freezing; Table 1). The median (IQR) TTD for all the 1G tests was 17 (7) days. All the conventional methods were done with sputum frozen once at −20 °C, with TTD shown in Table 2. Namely, the direct AFB sputum smear microscopy took 2 days. The MGIT-manual took 14 days for initial assessment of mycobacterial growth by AFB smear microscopy, plus an additional culture into LJ slants to confirm *Mtb* growth, resulting in a total turnaround time of approximately 42 days for detection, without DST results. The automated MGIT-960 required 7 ± 3 days for *Mtb* detection, and the additional DST required dilutions and subcultures into separate anti-TB drug–containing tubes, followed by incubation for 12 to 14 days for automated MGIT-960 system. Altogether this 2-step MGIT-DST protocol took 19 to 21 days from the time of initial sputum culture.

### Characteristics of Participants With DR-TB in Mexican Cities Across the Texas Border

The DST results were used to characterize the epidemiology patterns of DR-TB in our study population. We used the DST data from the 1G test given (1) the high concordance between the 1G test and the automated MGIT-960 (Table 3) and (2) the availability of DST data for all *Mtb*-positive 1G test results vs only a subset of drug-susceptible *Mtb* (n = 19) assessed by MGIT-DST. The DR-TB profiles are shown in Table 4. The prevalence of any DR-TB was 14% (52/376), with INH resistance at 11% and RIF or MFX at 4%. Monoresistance was 7% for INH, 0.3% for RIF, and 3% for MFX. MDR-TB was 3% and pre-extensively DR-TB (MDR plus MFX resistance) was 0.6%. Host characteristics were not associated with DR-TB (Supplementary Table 3).

**Table 4.** Prevalence of DR-TB in Patients With Pulmonary TB From Mexican Border Communities

	Drug Resistance, %^a^		
Type	No.	Among all *Mtb*, %	Among resistant *Mtb*, %
Any resistance			
INH-R	41	11	79
RIF-R	16	4	31
MFX-R	12	4	23
Monoresistance			
INH-R	27	7	52
RIF-R	1	0.3	2
MFX-R	9	3	17
Multidrug resistance			
INH/RIF	12	3	23
INH/RIF/MFX^b^	2	0.6	4
RIF/MFX	1	0.3	2
Total with any resistance	52	14	100

Prevalence based on DR-*Mtb* detected by the 1G test.

Abbreviations: DR, drug resistant; INH, isoniazid; MFX, moxifloxacin; *Mtb*, *Mycobacterium tuberculosis*; R, resistant; RIF, rifampicin; TB, tuberculosis.

^a^Denominator is 376 for RIF-R and INH-R and 310 for analysis containing MFX-R.

^b^Pre-extensively DR-TB.

## DISCUSSION

We evaluated the 1G test in TB clinics from small cities in the Mexican border with Texas, where DR-TB testing is not locally available and referrals to central clinics are limited to cases with high risk of DR-TB. We found that the 1G test was more sensitive for *Mtb* detection than the MGIT-manual (18 positive specimens by 1G but negative by MGIT-manual) but comparable to automated MGIT cultures and less prone to contamination. Moreover, the 1G test had good concordance with the MGIT-DST for INH, RIF, and MFX, with the added advantage of less contamination, faster results, and a simpler protocol. It also had similar TTD for *Mtb* but shorter for DST vs the automated MGIT-DST, and the test comprised a simple 1-step process as compared with additional supplies, personnel, and equipment for the MGIT-DST. Altogether, the 1G test is a low-complexity phenotypic TB diagnostics test that offers a practical alternative to phenotypic tests for *Mtb* detection and DST (eg, LJ or MGIT with DST). Its simplicity makes it suitable for use in decentralized laboratories in mid- and high-burden TB regions, where smear microscopy is routine, biosafety cabinets are available, and molecular testing is not feasible due to high cost and limited resources (eg, trained personnel, expensive instrumentation). Additionally, while molecular testing targets mutations conferring resistance, there remain DR-TB cases that can be detected only with phenotypic methods.

Our study setting shares the limitations for TB diagnosis as many other regions worldwide. Namely, Mexican TB referral clinics on the border with Texas do not offer routine testing for DR-TB, unless the individual is younger than 5 years, has treatment failure, is immunocompromised, or poses a high risk for DR-TB [21]. In some countries with high TB burden, Xpert (Cepheid) is replacing AFB smear microscopy testing, but in Mexico this technology is not subsidized, is costly (>$50/cartridge), and is not readily available [7]. Instead, DST is centralized and results can take up to 6 months [7]. Our results provide support for the value of the 1G test as a technically simple, reliable phenotypic method for diagnosis of TB and DR-TB in these types of settings and without need for additional infrastructure or biosafety considerations.

The 1G test had comparable performance when sputa were processed by the NALC-NaOH or SMD method. The use of SMD for digestion and decontamination plus the 1G test for *Mtb* isolation and DST testing has the advantage of requiring less sputum (150 μL) and can be performed without equipment. Namely, the SMD vortexing step can be replaced by handshaking or mixing via a disposable transfer pipet to avoid aerosol or froth generation [8, 12]. Furthermore, the 37 °C incubation can be done without CO_2_ or even at room temperature in countries where the ambient temperature is closer to 37 °C, although with a longer TTD (Torrelles, unpublished findings). However, further evidence is needed to determine the biosafety implications of these modifications, including whether personnel safety still requires the use of a biosafety cabinet.

The 1G test had a shorter turnaround time at 2 weeks and a simpler and economical 1-step protocol vs the 2-step MGIT and MGIT-DST protocols that require equipment and additional steps. The TTD of the 1G test was also shorter when compared to the standard 28 to 42 days required for *Mtb* detection alone with LJ cultures.

Despite good agreement between the 1G test and MGIT for DST, the nature of their discordant results deserves further evaluation. RIF showed the highest discrepancy. This may reflect RIF instability during the 42-day 1G incubation or the inability of the MGIT-DST to detect low-level RIF resistance in some strains, considering the recent WHO recommendation to lower the MGIT-DST RIF critical concentration from 1.0 to 0.5 mg/L [22–26]. This lower concentration is already used in the 1G test. Understanding the molecular basis for discrepancies is clinically relevant given associations between low-level RIF resistance and poor treatment outcomes [26]. Genotyping is in progress to clarify if discordant findings represent false-positive results.

DR-TB was estimated at 6% in northern Tamaulipas in 2020 by passive reporting, while our results between 2020 and 2024 suggested that DR-TB is more than twice as high (14%). The passive underestimation is likely due to the lack of routine DST testing, although sampling bias in our study cannot be excluded. The MDR-TB rate of 3% in our study population is comparable to 3.2% globally [1]. The 4% prevalence of RIF resistance with the 1G test was below the global estimate of 6.9% and was even lower when determined by the MGIT-DST [27]. Only 2 of 14 (14%) MDR-TB cases had additional resistance to MFX (pre-extensively DR-TB), which is lower than the reported WHO rate of 20% [1].

Detection of MFX-resistant isolates was unexpected, as this drug has not been introduced for TB treatment locally. A possible explanation is cross-resistance due to the unprescribed use of fluoroquinolones in our study population [28]. The WHO recommends MFX for treating drug-susceptible TB, INH-monoresistant TB, and MDR-TB, but our finding points the need for MFX DST prior to its use in patients with TB at the Mexican border [29]. Unlike previous studies, we found no association between DR-TB and host factors [30], which may reflect the small sample number of DR-TB cases in our study.

A study limitation is the lack of simultaneous testing with the 1G test and conventional methods, which required an additional freezing step for two-thirds of the samples tested with the 1G test. Despite this disadvantage, the 1G test demonstrated a robust performance except for a median 3-day delay in TTD. Even though there was perfect concordance between the 1G test and MGIT-DST for a subset of pan-sensitive *Mtb* isolates identified by the 1G test, we cannot rule out missing DR-*Mtb* isolates unique to the MGIT-DST. The prevalence of RIF resistance in our community based on the 1G test should be interpreted with caution given its higher prevalence vs MGIT-DST. Most study participants had a positive AFB sputum smear microscopy, and our conclusions should take into consideration this potential bias.

In conclusion, our findings build on previous work to provide support for the 1G test as an accurate, simple, and affordable alternative to current phenotypic methods for TB detection in resource-limited high-burden settings. Used in parallel with sputum smear microscopy at centralized or decentralized clinics, it can enhance mycobacterial detection sensitivity and enable DST, especially where molecular tools such as GeneXpert are not subsidized. Future studies are warranted to evaluate the implementation of the 1G test in decentralized clinics lacking molecular diagnostic capacity, with simple modifications as needed, such as the use of SMD, transfer pipets for mixing with minimal aerosol generation, and incubations at room temperature.

## Supplementary Material

jiaf638_Supplementary_Data

## Supplementary Data

Supplementary materials are available at *The Journal of Infectious Diseases* online (http://jid.oxfordjournals.org/). Supplementary materials consist of data provided by the author that are published to benefit the reader. The posted materials are not copyedited. The contents of all supplementary data are the sole responsibility of the authors. Questions or messages regarding errors should be addressed to the author.
