# Supplementary material for: Accuracy of the phenotypic 1G test to detect Mycobacterium tuberculosis and drug resistance from sputa in the US-Mexico border
Source: J Infect Dis. Author manuscript; Available in PMC 2026 Jan 30. (PMC12853293; doi:10.1093/infdis/jiaf638)
Supplement: Supplementary file 1 [file NIHMS2133382-supplement-Supplementary_Material.docx]

**SUPPLEMENTAL MATERIAL**

**Manuscript: Accuracy of the phenotypic 1G test to detect** ***Mycobacterium tuberculosis*** **and drug resistance from sputa in the US-Mexico border**

Mia Aguirre, Doris Ayala, Juan Ignacio Garcia, Yoscelina E. Martinez-Lopez, Amberlee D. Hicks, Nadine Chacon, Ashley Gay-Cobb, Alyssa Schami, Selena Zavala-Perez, Ilse A. Dominguez-Trejo, America M. Cruz-Gonzalez, Raul Loera-Salazar, Javier E. Rodríguez-Herrera, Esperanza M. Garcia-Oropesa, Miryoung Lee, Adrian Rendón, Shu-Hua Wang, Marcel Yotebieng, Carlton A. Evans, Jordi B. Torrelles, Blanca I. Restrepo

**TABLE OF CONTENTS**

**Supplement 1. Algorithm for sputum specimen processing protocols ……………………………………………….……..1**

**Supplement 2. Manual MGIT protocol for** ***Mycobacterium tuberculosis*** **(*****Mtb*****) detection ……………………….………..2**

**Table S1. Reference strains and the corresponding resistance ……………………………………………………………..2**

**Table S2. Characteristics of study participants evaluated for TB using the 1G test (N=426) ……………………..……..3**

**Table S3. Host characteristics associated with DR-TB ……………………………………………………………………..4**

**Figure S1. Sputum sample algorithm for data analysis of the 1G test …………………………………………………….5**

**Supplement 1. Algorithm for sputum specimen processing protocols**

A total of 426 possible TB patients were sequentially enrolled between June 2020 and September 2024 at reference outpatient TB clinics operated by the Secretaría de Salud of Tamaulipas, Mexico, and their sputa processed with the NALC-NaOH protocol and evaluated for the *Mtb* detection using standard methods: AFB smear microscopy and MGIT cultures (Fig. 1). The assessment of the 1G test for *Mtb* detection began in November 2021. A subset of samples was processed using an alternative salt-mix decontamination (**SMD**) method that does not require centrifugation, with the goal of comparing it to the NALC-NaOH protocol. ^1,2^ Selection of specimens for this processing method was as follows: For sputa already evaluated by conventional phenotypic methods, thawed sputum *N*-Acetyl-L-Cysteine and 2% sodium hydroxide (NALC-NaOH, Hardy Diagnostics, Santa Maria, CA) decontaminated aliquots were used (n=302). When these were unavailable, leftover raw sputum aliquots were thawed and processed using the SMD method (n=28). Namely, sputum was mixed with SMD in a 1:2 ratio (2x SMD stock solution: 2 g tri-sodium phosphate, 0.05 g ammonium sulphate, 0.005 g magnesium sulphate, 0.0025 g ferric ammonium citrate, 10 mL sterile water, and 0.01 mL red food coloring, and 100 IU/ml of penicillin), vortexed briefly, and incubated at room temperature for 10 min to 1 hour. For newly collected specimens, some were treated with NALC-NaOH only (n=61), while others were processed in parallel using both NALC-NaOH and SMD (n=35) for comparative purposes (Fig 1). 

**References:**

1.	Zhang A, Jumbe E, Krysiak R, et al. Low-cost diagnostic test for susceptible and drug-resistant tuberculosis in rural Malawi. *Afr J Lab Med* 2018; **7**(1): 690.

2.	Evans C. Innovation for Health and Development: Colour test. 2021 2021. [https://www.ifhad.org/colour-test/2025](https://www.ifhad.org/colour-test/2025)).

**Supplement 2. Manual MGIT protocol for** ***Mycobacterium tuberculosis*** **(*****Mtb*****) detection**

Five hundred microliters of sputum treated with NALC-NaOH were added into a 4-mL Mycobacteria Growth Indicator Tube (MGIT) and incubated at 37°C in 5% CO_2_. After one week, 1 mL was pelleted, inoculated onto solid Löwenstein–Jensen (LJ) medium Gruft slants (Hardy Diagnostics) and incubated at 37°C with 5% CO_2_ for detection of *Mtb* growth for up to 42 days. The remaining MGIT media culture was incubated for an additional week and the sediment evaluated for AFB smear microscopy. A manual MGIT test was considered positive for mycobacteria when the MGIT broth pellet or LJ growth were positive by AFB smear microscopy.

| **Table S1. Reference strains and the corresponding resistance** |  |  |  |  |  |  |
| --- | --- | --- | --- | --- | --- | --- |
| **Strain** | **STR** | **INH** | **RIF** | **EMB** | **FQL (MFX)** |  |
| W 565 | R (rpsL/rrs*) | R (katG*) | R (rpoB*) | R (embB*) | R (gyrA*) |  |
| AH 32300 | R (rpsL/rrs*) | R (katG/inhA*) | R (rpoB*) | - | R (gyrA*) |  |
| MH 32216 | R (rrs*) | R (katG/inhA*) | R (rpoB*) | - | R (gyrA*) |  |
| HP134 31011 | R (rrs*) | R (katG/inhA*) | R (rpoB*) | R (embB*) | R (gyrA*) |  |
| *Gene with mutation conferring antibiotic resistance. |  |  |  |  |  |  |
| Abbreviations: R=resistant; STR=streptomycin; INH=isoniazid; RIF=rifampicin; EMB=ethambutol; FQL=flurpquilonone; MFX=moxifloxacin; rpsL=ribosomal S12 protein; rrs=ribosome biogenesis regulator 1 homolog; katG=catalase-peroxidase enzyme; inhA=inhibin Alpha; rpoB=B subunit of RNA polymerase; embB=EmbB protein; gyrA=DNA gyrase enzyme |  |  |  |  |  |  |
|  |  |  |  |  |  |  |

**Table S2. Characteristics of study participants evaluated for TB using the 1G test (N=426)** 

| **Variables (n with data)** | **n (%) or median (IQR)** |
| --- | --- |
| **Sociodemographic characteristics** |  |
| Hispanic white | 426 (100%) |
| Age, years (IQR; range) | 43 (26; 28-54) |
| Male sex | 294 (69%) |
| BCG vaccine | 392 (92%) |
| Marital status married/cohabitation | 261 (61%) |
| Education up to middle school | 294 (69%) |
| Current smoker ^a^ | 20 (5%) |
| Alcohol abuse | 73 (17%) |
| Frequent drug use (weekly or daily) ^b^ | 87 (21%) |
| **Medical conditions** |  |
| High blood pressure | 92 (22%) |
| Positive HIV serology | 25 (6%) |
| Diabetes and pre-diabetes classification |  |
| No diabetes | 143 (34%) |
| Pre-diabetes | 95 (22%) |
| Diabetes | 188 (44%) |
| Years with diabetes ^c^ | 0 (5) |
| **Past TB history** |  |
| Past TB history | 70 (16%) |
| *Time since last episode among past TB* |  |
| Less than 2 years ago ^d^ | 31 (44%) |
| More than 2 years ago ^d^ | 39 (56%) |

BCG, Bacillus Calmette-Guerin; IQR, inter-quartile range. 

^a^: n=424, ^b^: n=423, ^c^: n=188, ^d^: n=70

**Table S3. Host characteristics associated with DR-TB** ^**1**^

| **Variables** | **Any DR**  **n=52** | **Pansensitive**  **n=323** | **p-value**^**2**^ |
| --- | --- | --- | --- |
| **Sociodemographic characteristics** |  |  |  |
| Age, years (IQR; range) | 36 (22; 26 - 48) | 43 (25; 29 - 54) | 0.091 |
| Male sex | 39 (74%) | 218 (67%) | 0.377 |
| BCG vaccine | 47 (89%) | 305 (94%) | 0.113 |
| Marital status married/cohabitation | 28 (53%) | 208 (64%) | 0.250 |
| Education up to Middle School | 35 (66%) | 222 (69%) | 0.696 |
| Current smoker | 3 (6%) | 13 (4%) | 0.734 |
| Alcohol abuse | 3 (6%) | 12 (4%) | 0.502 |
| Frequent drug use (weekly or daily) | 12 (23%) | 66 (21%) | 0.579 |
| **Medical conditions** |  |  |  |
| Positive HIV serology | 2 (4%) | 14 (4%) | 0.851 |
| High blood pressure | 12 (23%) | 69 (21%) | 0.834 |
| Diabetes and pre-diabetes classification |  |  |  |
| No diabetes | 24 (45%) | 102 (32%) | 0.109 |
| Pre-diabetes | 7 (13%) | 70 (22%) |  |
| Diabetes | 22 (42%) | 151 (47%) |  |
| **Past history of TB** |  |  |  |
| Past history of TB | 7 (13%) | 51 (16%) | 0.629 |
| *Time since past TB diagnosis* ^*3*^ |  |  |  |
| Less than 2 years ago | 4 (8%) | 23 (8%) | 0.109 |
| More than 2 years ago | 3 (6%) | 28 (10%) |  |

^1^ Data expressed as n (row %), where the denominator for individuals with past TB is 376 participants. ^2^ Comparison between continuous variables by Wilcoxon rank-sum and categorical by chi-squared or Fisher’s exact when cells had 5 or less counts. P-values in bold is for significant or borderline significant. 

**Figure S1. Sputum sample algorithm for data analysis of the 1G test**. ^1^ n=376 evaluated with the 1G test for INH- and RIF-DR, and n=310 for MFX; Gray background, specimens excluded from data analysis.
